# Evidence of an ‘invitation’ effect in feeding sylvatic *Stegomyia albopicta* from Cambodia

**DOI:** 10.1186/1756-3305-7-324

**Published:** 2014-07-11

**Authors:** J Derek Charlwood, Elsa VE Tomás, Louise Kelly-Hope, Olivier JT Briët

**Affiliations:** 1 Vector Group, Liverpool School of Tropical Medicine, Pembroke Place, Liverpool L3 5QA, UK; 2 MOZDAN (Mozambican-Danish Rural Malaria Project), PO Box 8, Morrumbene, Inhambane Province, Mozambique; 3 Centre for Neglected Tropical Diseases, Liverpool School of Tropical Medicine, Pembroke Place, Liverpool L3 5QA, UK; 4 Swiss Tropical and Public Health Institute, Socinstrasse 57, PO Box, CH-4002, Basel, Switzerland; 5 University of Basel, PO Box, CH-4003, Basel, Switzerland

**Keywords:** Invitation effect, Body site preference, *Stegomyia albopicta*, Landing, Cambodia

## Abstract

**Background:**

Orientation of haematophagous insects towards a potential host is largely mediated by kairomones that, in some groups or species may include chemicals produced during feeding by the insects themselves, the so called ‘invitation’ effect.

**Methods:**

The ‘invitation’ effect in blood-feeding diurnally active *Stegomyia albopicta* was investigated over 33 days in secondary forest in Mondolkiri Province, Cambodia. Two human volunteers sitting inside a shelter collected mosquitoes and noted where and when they landed. A 10% emanator of a synthetic pyrethroid with high vapour action was in use on alternate days.

**Results:**

Overall, 2726 mosquitoes were collected, 1654 of which had the landing site recorded. The heads of the volunteers were the locations with the highest density of landings per surface area whilst the knees and elbows accounted for most of the landings received on the arms and legs. Landings recorded within three minutes of each other on a collector were about 2.5 times more likely to be on the same body part than on a random body part, weighted for landing site preference. This preference did not vary with collector or pyrethroid.

**Conclusions:**

The ‘invitation’ effect may be due to a semio-chemical produced early in the feeding process. Incorporation of such a chemical into traps designed to control this important vector of dengue and chikungunya viruses might potentially improve their attractiveness.

## Background

Many haematophagous insects have a preference to feed on a particular site on the body of their hosts, which varies from the ankles in *Anopheles farauti* and *Anopheles gambiae* sensu lato [1,2] to the head in *Anopheles atroparvus*[2] and even the nose in *Sabethes belisarioi*[3]. To date, however, studies of mosquito landing on hosts have largely focused on the number of insects biting a host rather than on the site on the host where they feed. Although orientation of mosquitoes and other haematophagous insects towards a potential host or body part is largely mediated by host kairomones and other factors such as body heat, in some groups or species, chemicals produced or released by the feeding insects themselves may act as attractants. The so-called ‘invitation’ effect was originally described by Alekseev and colleagues [4], who found that more *Aedes communis* are attracted to the arm of a collector with 50 or 100 *Ae. communis* confined in a cage feeding on it, compared to a control arm without mosquitoes. Female *Aedes sierrensis* are also more likely to enter a chamber emanating air from feeding conspecific females or from female *Stegomyia aegypti*, also known as *Aedes aegypti,* than a control chamber from which the air immediately surrounding the feeding females was diverted [5]. Similarly, Cavanagh and Townson [6] found that while significantly greater numbers of mosquitoes are attracted to an ‘artificial host’ (chicken skin on a membrane feeding apparatus) on which mosquitoes are feeding than to the artificial host alone, mosquitoes that feed through a plastic membrane fail to elicit the response, which suggests that host odour acts as a releaser which sensitizes host seeking mosquitoes to a chemical released by the feeding mosquitoes. *Aedes cantans* are also more likely to attempt to feed on a leg that has a cage of feeding conspecifics strapped to it than on the control leg with conspecifics in a similar cage that are prevented from feeding [7]. No such effect has been observed, however, using the same experimental technique, primarily among anopheline mosquitoes from East Africa, which the authors attribute to the fact that there was a mixture of species feeding and that the latter experiments were performed at the insect’s usual activity time [7]. Apart from effects observed in *Aedes* spp., invitation effects in blood-feeding flies particularly occur among insects that pool feed, such as sandflies [8]. Such group feeders are known to produce a pheromone to attract other hungry females. When confined in small cages, *Simulium damnosum* also appear to be more likely to feed when others are doing so [9], whilst parous females of the Scottish midge *Culicoides impunctatus* also appear to attract others to a host when they are feeding [10].

Little is known about the landing preference of the Asian tiger mosquito, *Stegomyia albopicta* (also known as *Aedes albopictus*) despite the fact that it is an important vector of dengue and chikungunya viruses. The mosquito, which is largely sylvatic, differs from *St. aegypti* in that it tends to take a full blood meal rather than a series of partial feeds in each gonotrophic cycle [11]. Compared to *St. aegypti*, which is the most important vector of dengue, *St. albopicta* is a less competent vector of arboviruses, and, perhaps because of this feeding difference, the epidemics it causes are milder. It is less anthropophilic and is not as well adapted to urban domestic environments as *St aegypti*. However, it easily adapts to new environments, even temperate ones, so that, largely due to the trade in used car tyres (which provide a suitable environment for larval development), its distribution is rapidly expanding and its importance is increasing.

Should *St. albopicta* have a strong preference for any particular part of the body, protecting these areas may help to reduce disease transmission. Whether or not feeding *St. albopicta* produce ‘invitation’ effects is also unknown. The presence of a large population of hungry sylvatic *St. albopicta* mosquitoes and their unavoidable attacks on two hosts provided an opportunity to examine the existence of landing site preference and ‘invitation’ effects in this species.

## Methods

During a project to investigate ways to reduce malaria transmission in Cambodia, JDC, a 63 year old, 72 kilogram 1.68 m, male, with skin type III on the Fitzpatrick scale [12], and EVET, a 30 year old, 64 kilogram 1.75 m, female with skin type VI on the Fitzpatrick scale, camped in secondary forest close to the village of Ou Chrar in Mondolkiri Province, Cambodia (N 12° 14’ 484”, E106° 50’ 945”) from the 20th of April to the 2nd of June 2013.

**Figure 1:**
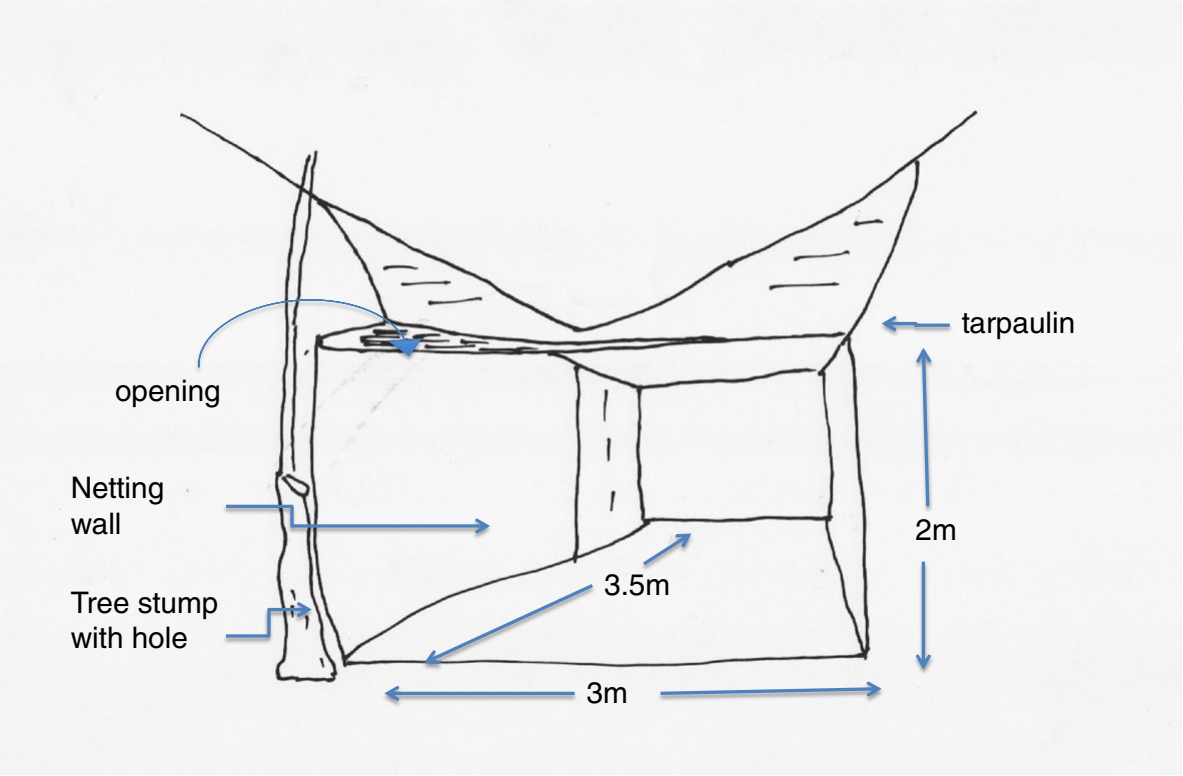
Sketch of the experimental shelter showing the location and size of the opening through which mosquitoes entered during the collections, Ou Chrar woods, Mondolkiri Province, Cambodia.

**Figure 2:**
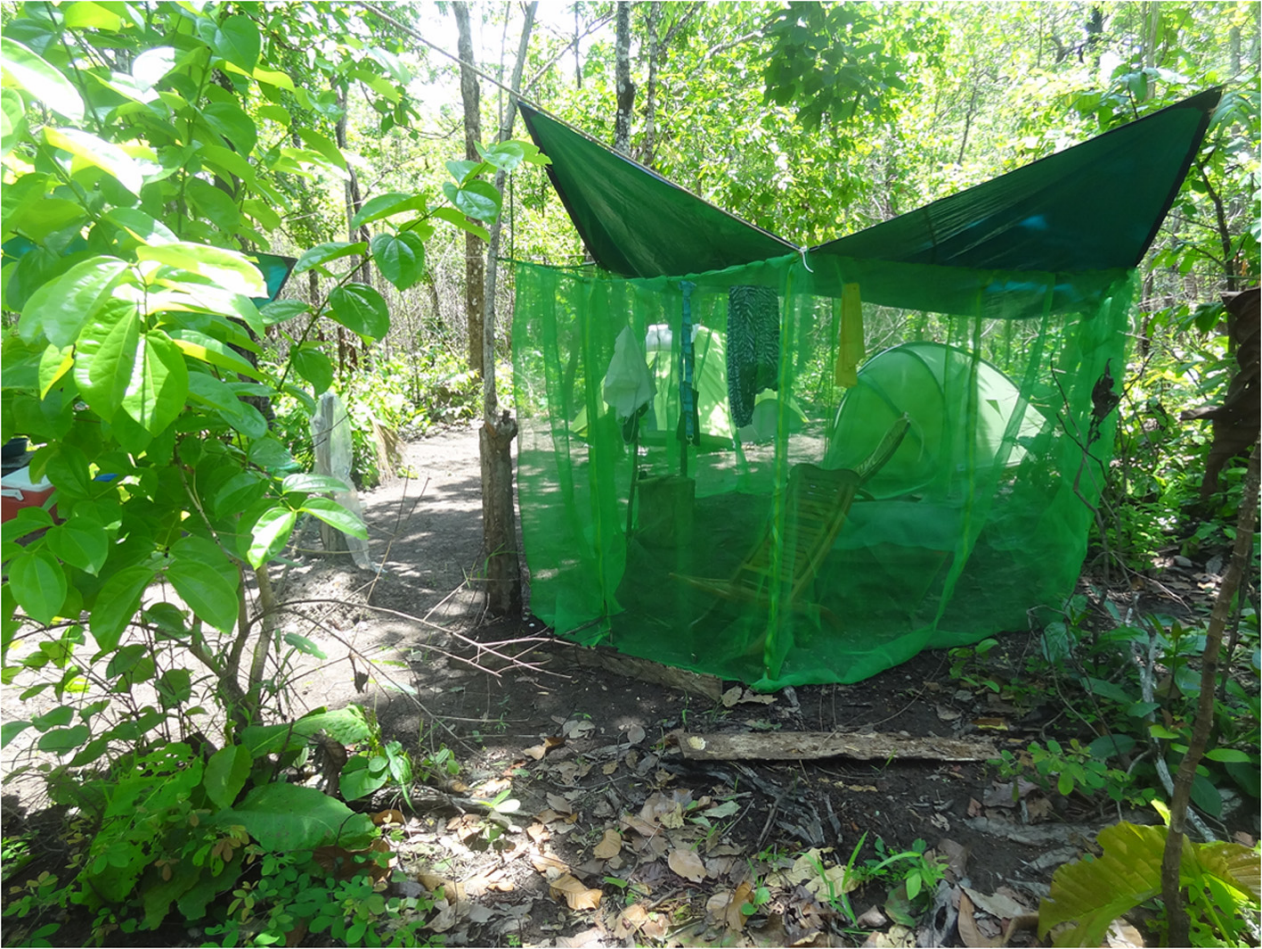
Photograph of the rear of the experimental shelter.

**Figure 3:**
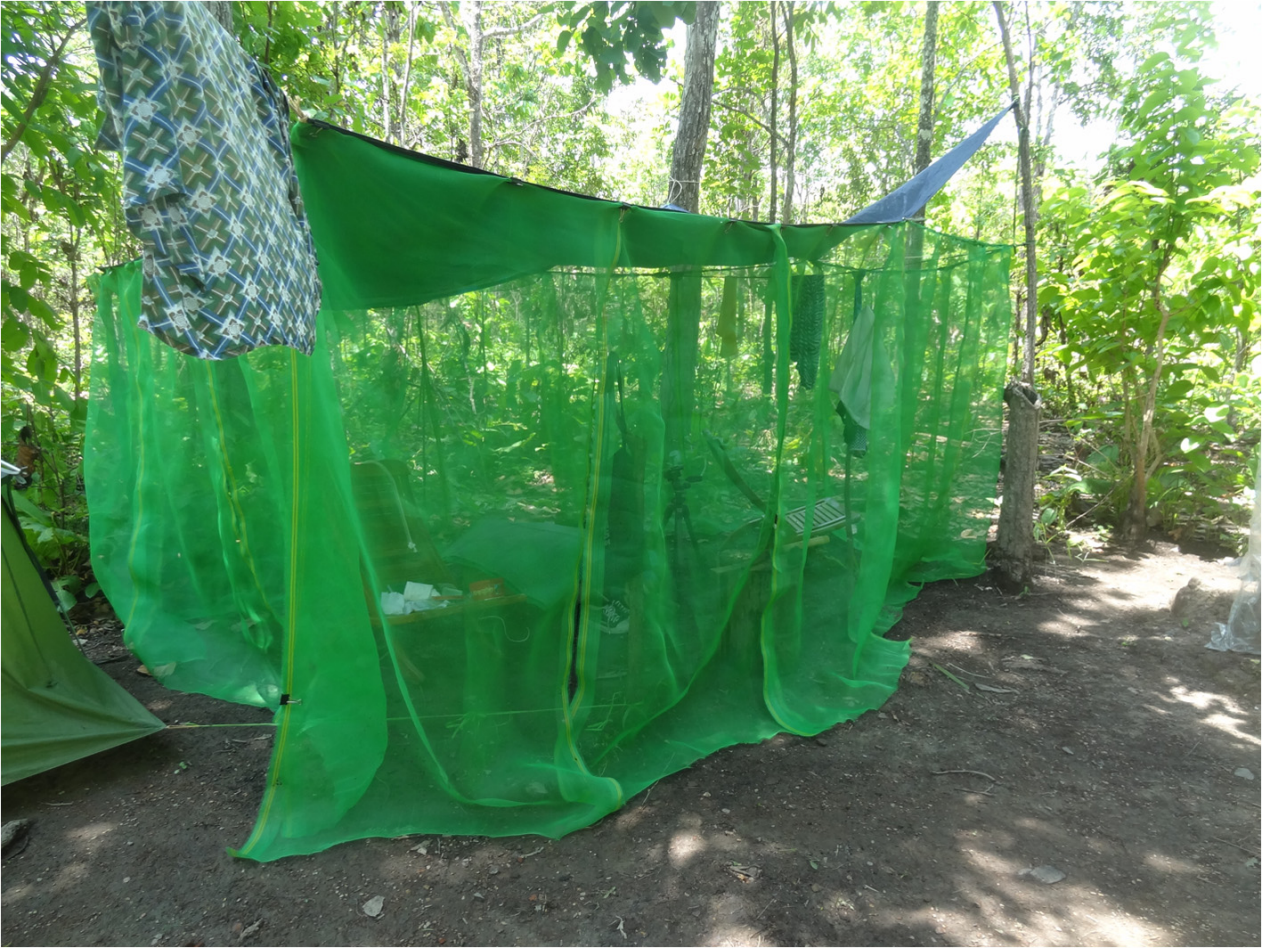
Photograph of the front of the experimental shelter.

Most of the trees in the forest had a girth of less than 30 cm and there was only limited undergrowth. Water-filled tree holes provided a suitable habitat for mosquito larvae. In order to avoid the annoyance of sweat bees (*Halictidae*), a variety of horseflies (*Tabanidae*) and day biting mosquitoes, they constructed a 2x3x4 m (24 m^3^) shelter of 1.3 m wide overlapping plastic mosquito netting strips with a tarpaulin roof for their daytime living area. All but two of the strips were tied together with thin wire to make a sealed wall. The two remaining strips could be raised and lowered and acted as a door. The door of the shelter was left open from nightfall to midday at which time it was closed. Between noon and nightfall the strips were lowered and the shelter effectively closed. There was, however, a horizontal opening at the height of the wall, in the form of an isosceles triangular extension of the walls away from the roof at one end of the shelter (Figures 1, 2 and 3). Despite this opening, the entry of the bees and horseflies was curtailed. Mosquitoes, however, continued to enter the shelter through this opening. The half of the shelter where landing collections were performed had a floor of yellow and white empty polythene rice sacks. On alternate days, a slow-release emanator made of polyethylene mesh impregnated with a high vapour action pyrethroid was suspended in the shelter close to the opening.

One or both campers performed *ad hoc* landing collections before noon, attempting to collect mosquitoes landing on themselves before the mosquitoes fed. From 12:00 until 17:30 or until 17:00 for 33 of 35 days between the 28 April and the 30 May 2013, both campers, both wearing shorts and lightweight shirts or T-shirts, sat on wooden reclining chairs and conducted controlled landing and resting collections on themselves and each other, sitting opposite each other close to the open end of the shelter which was in the shade away from direct sunlight. By sitting upright on the chairs, which they did most of the time, the only parts of the body unavailable for mosquitoes to land on were their scalp, groin, buttocks and soles of their feet. EVET also periodically searched the inside walls of the shelter for mosquitoes. On three days, collections started at 14:00, and on five days, collections stopped at 17:00. For the first 16 days of collection, the two collectors exchanged place every second day (i.e. they collected in the same place for two days before changing places. Thus they collected in the same place once during a control collection and once when the pyrethroid was in use. From the 17th day of collection until the end of the study (thus for the last 17 days of the study), for practical reasons, they collected from the same place every day (JDC closer to the opening and pyrethroid when it was in use and EVET circa 1.7 m distant). From the 3rd of May to the 3rd of June the time of landing (to the minute), the host and the site where the mosquito landed were noted. For lateral sites, the side of landing on the body (left or right) was, however, only recorded when a mosquito attempted to feed on the same body part on the opposite side of the body within three minutes of a previously collected mosquito.

After collection, mosquitoes were grouped in cups and later stored in eppendorf tubes over silica gel. Mosquitoes were identified to sex and, for females, abdominal condition (fed or unfed) when collected and, subsequently as *Stegomyia albopicta* or non-*Stegomyia* species using a stereo-microscope on site. A sample of stored mosquitoes was later identified using the keys of Rattanarithikul and colleagues [13] in Liverpool.

Temperature and humidity were recorded on an hourly basis using a Davis Weathervue weather station whilst airflow was measured, with the door open and closed, using a TG440 Airflow meter placed within 10 cm of the pyrethroid dispenser. Daily rainfall was recorded in the village 700 m from the collection site.

In order to investigate the invitation effect in a statistical procedure [Additional file 1], if the side of landing (left or right) was not specified in the data for a lateral body part, each *Stegomyia* mosquito’s landing on a lateral body part was randomly assigned a side. Subsequent mosquito landings within three minutes of the previous mosquito on the same person and lateral body part were thus assigned to the same side, unless the data indicated that the subsequent landing was on the opposite side. The total number of subsequent landings on the same body part within three minutes was scored per collector and per day. Then, for each day for each collector, the body part of landing was permutated randomly (while keeping the landing times fixed), and again the total number of landings on the same body part within three minutes was scored. This procedure was repeated 1000 times, yielding a distribution for the observed score, and a distribution for randomized landing locations. Subsequently, these distributions were compared.

Landings on 34 different parts of the body per person were recorded. Because some of these were ambiguous (e.g. the recorded landing site ‘head’ existing alongside more exact descriptions such as ‘forehead’), these were merged into eight body regions. The body surface area of both collectors was calculated according to the formula of Du Bois and Du Bois [14].

### Ethical statement

The collections described in this article form part of the ‘Artemisinin Resistant Malaria Research Programme - Assessment of Novel Vector Control Interventions’ which was approved by the ethical committees of the National Centre of Malariology (CNM) in Phnom Penh, (Cambodia) and of the Liverpool School of Tropical Medicine (UK). Collections were performed by JDC and EVET. Both had access to medical treatment.

## Results

A total of 2905 female mosquitoes, 855 on JDC, 1054 on EVET and 996 from the walls of the shelter, were collected [Additional file 2]. Despite close attention, many mosquitoes managed to feed as evidenced by the numbers of engorged mosquitoes that were also collected off the inside netting walls of the shelter. Most of these blood-fed females were in the vicinity of the hosts and most of them were seen as, or shortly after, they landed on the walls. Most (98.2%) of the mosquitoes were *St. albopicta* although specimens of *Armigeres kesseli*, *Arm. (Lei.) annulipalpis*, *Zeugnomyia gracilis* and a small number of unidentified culicines were also collected.

**Figure 4:**
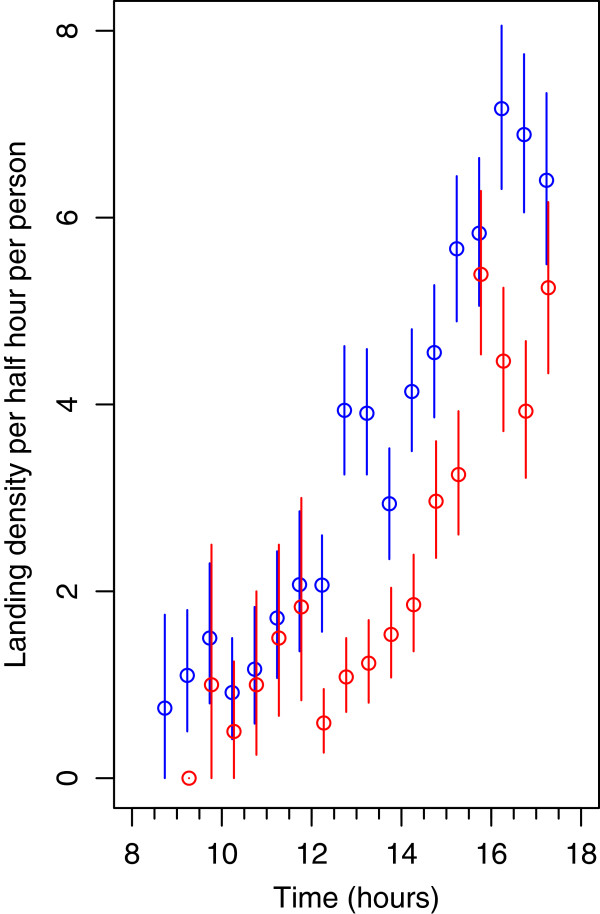
**Landing activity of *St. albopicta *between 08:30 and 17:30 in Ou Chrar woods, Mondolkiri Province, Cambodia May – June 2013.** Vertical lines indicate 95% confidence intervals. Red: With a pyrethroid emanator Blue: Without emanator

Numbers of *St. albopicta* biting increased to a maximum between 15:30 and 16:30 (Figure 4). The mean temperature recorded at 16:00 (the time of peak biting) was 32.6°C (minimum 32°C, maximum 36°C) and the mean relative humidity was 75.4% (minimum 60%, maximum 87%). Rain fell on 29 of the 40 days between the 21st of April and the 30th of May. On 11 of these days, more than 20 mm of rain was recorded. Over the entire study period, a total of 353.7 mm of rain was recorded in the village. Most of the rain fell during the night or late evening. However, on four days, heavy rain fell during the period between 14:00 and 17:00. This significantly reduced biting (p value <0.0001) according to the exact rate ratio test and assuming Poisson counts; thus only 53.4% (95% C.I. = 44.8 – 63.0) of the expected numbers were collected on days when heavy rain fell during the period between 14:00 and 17:00 as compared to when there was no heavy rain. Airflow was always low: with the door open, it was generally less than 0.25 m per second, while closing the shelter doors reduced airflow by approximately 50%.

**Figure 5:**
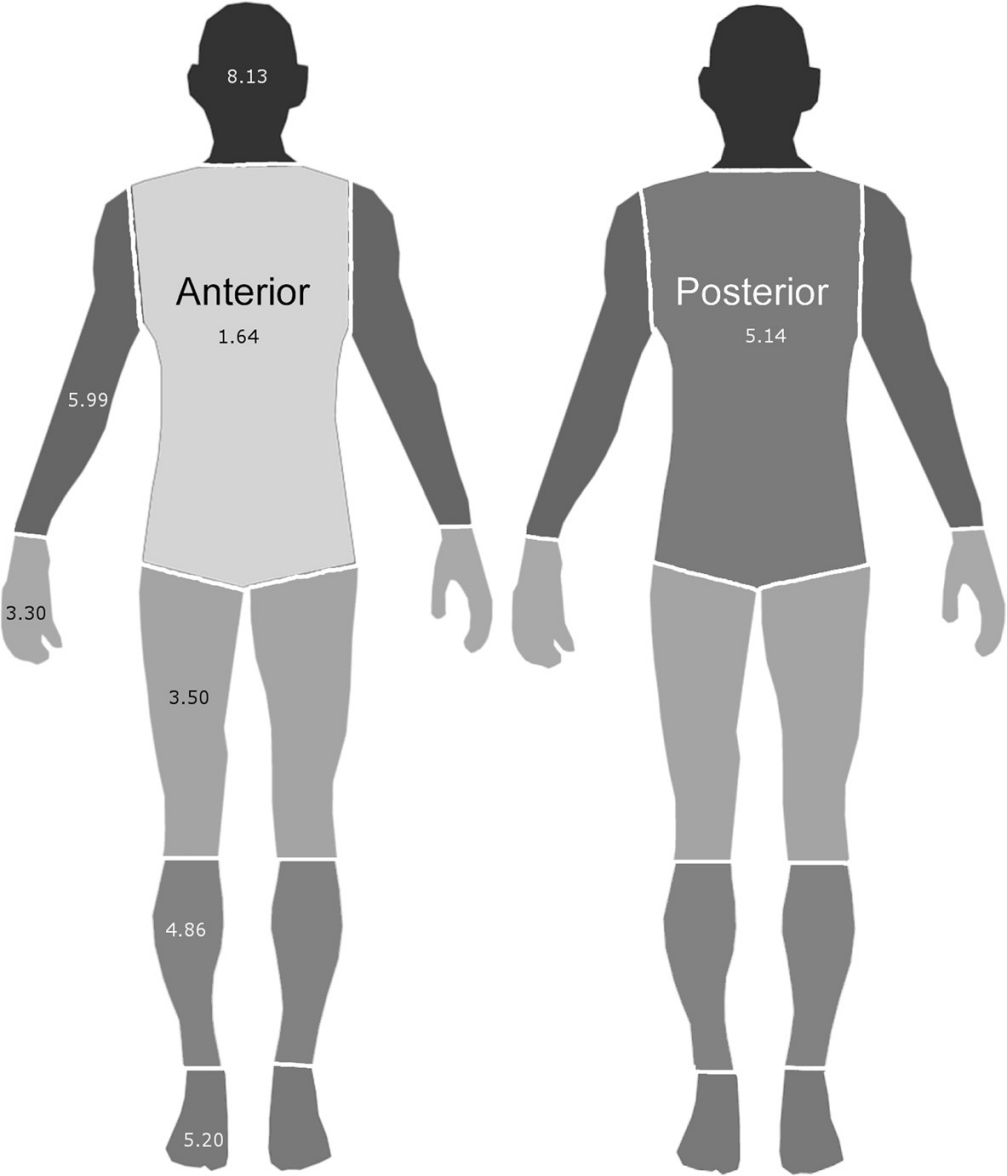
**Average landing density (dm**^**-2**^**) on body regions.**

The estimated body surface area of the collectors was similar (1.78 m^2^ and 1.82 m^2^ for EVET and JDC, respectively). For each dm^2^ of body surface, over the 191.5 hours of collecting per person, EVET recorded 5.92 landings compared to 4.70 landings recorded by JDC. Thus, overall more mosquitoes were collected landing on EVET than on JDC (rate ratio =1.26, 95% CI: 1.15 – 1.39, Poisson-test p < 0.001). Stratified by collection period, the difference was significant in the first 16 days of collection when the collectors alternated between sides (rate ratio =1.73, 95% CI: 1.49 – 2.00, Poisson-test p < 0.001) but was not significant during the last 16 days when they did not (when JDC sat slightly closer to the widest part of the opening over which the mosquitoes presumably entered the shelter) (rate ratio =1.00, 95% CI: 0.89 – 1.13, Poisson-test p > 0.05). A total of 1654 *St. albopicta* were collected with the body part they landed on recorded, while for 252 other *St. albopicta* collected at the start of the study, the landing site on the body was not recorded (Table 1). These were excluded from subsequent analyses. Also, the 948 engorged mosquitoes collected from the walls were excluded. The distribution of the mosquitoes landing on the body regions were significantly different for both collectors (Pearson's Chi-squared test, p < 0.01, df = 7) with relatively more mosquitoes landing on JDC’s anterior trunk and relatively more mosquitoes landing on EVET’s hands (Table 2).Relative to surface area, most mosquitoes showed a preference for landing on the head (Figure 5). Since the estimation of surface area of the head included the scalp (which accounts for about half of the surface and, with the exception of JDC’s bald patch, was covered by hair preventing mosquitoes from landing) the preference for landing on the head was probably even greater. The cheeks (73 landings) and forehead (52 landings) accounted for 23% and 20%, respectively of the landings recorded on the head and neck, the remainder being on the neck (51 landings; 20%), ear (31 landings; 12%), nose (18 landings; 7%), chin, eye and eyebrow (10, 2 and 16 landings respectively). The knees accounted for most (37%) of the landings on the legs (excluding feet and ankles), and the elbows accounted for most (40%) of the landings on the arms.

**Table 1:** Distribution of landing sites of day biting mosquitoes collected from the two hosts during the study

**Body region**	**Body part**	**JDC**	**EVET**	**Wall**	**Sum**
Not available	Not available	109	143	948	1200
**Head**	Head	2	2		4
	Face		1		1
	Forehead	31	21		52
	Eyebrow	5	11		16
	Eye	1	1		2
	Nose	3	15		18
	Ear	4	27		31
	Lip	2	3		5
	Cheek	32	41		73
	Chin	5	5		10
	Neck	26	25		51
**Posterior trunk**	Back	35	10		45
	Shoulder	127	157		284
	Bottom		4		4
**Anterior trunk**	Chest	12	10		22
	Torso	37	18		55
	Waist	6			6
**Arms**	Arm	2	8		10
	Armpit		3		3
	Upper arm	31	76		107
	Elbow	50	71		121
	Forearm	21	39		60
**Hands**	Hand	7	22		29
	Wrist	4	26		30
**Upper legs**	Thigh	14	46		60
	Knee	103	76		179
**Lower legs**	Leg	10	20		30
	Lower leg	14	25		39
	Shin	24	8		32
	Calf	73	71		144
**Feet**	Ankle	23	32		55
	Foot	33	30		63
	Heel	5	4		9
	Toe	2	2		4
**Total**		**853**	**1053**	**948**	**2854**

Note that the insects collected without location information were collected at the start of the study.

**Table 2:** Landing density calculations

**Body region**	**Landings (95% CI)**		**Surface (cm**^**2**^**)**		**Landing density (dm**^**-2**^**)**		
	**JDC**	**EVET**	**JDC**	**EVET**	**JDC**	**EVET**	**Average**
Head	111 (91 – 132)	152 (128 – 177)	1638	1602	6.78	9.49	8.13
Anterior trunk	55 (41 – 70)	28 (18 – 39)	2548	2492	2.16	1.12	1.64
Posterior trunk	162 (138 – 187)	171 (146 – 197)	3276	3204	4.95	5.34	5.14
Arms	104 (85 – 124)	197 (170 – 225)	2548	2492	4.08	7.91	5.99
Hands	11 (5 – 18)	48 (35 – 62)	910	890	1.21	5.39	3.30
Upper legs	117 (96 – 139)	122 (101 – 144)	3458	3382	3.38	3.61	3.50
Lower legs	121 (100 – 143)	124 (103 – 146)	2548	2492	4.75	4.98	4.86
Feet	63 (48 – 79)	68 (52 – 85)	1274	1246	4.95	5.46	5.20

Subsequent *St. albopicta* recorded landings (within three minutes of each other) on a collector were about 2.5 times (Table 3) more likely to be on the same body part than on a random body part, weighted for landing site preference. This preference did not vary with collector or pyrethroid. With body parts aggregated into eight body regions (Table 4), the observed score was still significantly different from the score under randomization (p < 0.01).

**Table 3:** Ratio of scores for observed and randomized distributions of consecutive landings within three minutes on the same body part

**Collector / treatment**	**Mean (95% CI)**
All	2.48 (1.94 – 3.28)
JDC	2.52 (1.71 – 3.87)
EVET	2.55 (1.79 – 3.89)
Pyrethroid	2.73 (1.42 – 5.40)
Control	2.50 (1.84 – 3.37)

**Table 4:** Ratio of scores for observed and randomized distributions of consecutive landings within three minutes on the same body part, with body parts aggregate into eight regions, of which five were lateral body regions

**Collector/treatment**	**Mean (95% CI)**
All	1.78 (1.50 – 2.11)
JDC	1.86 (1.46 – 2.45)
EVET	1.74 (1.40 – 2.17)
Pyrethroid	1.61 (1.12 – 2.29)
Control	1.84 (1.53 – 2.24)

In a sample of 294 *St. albopicta* from eight days of landing collection, 228 (78%) had no discernible blood in their abdomen, thus they were largely collected within a short while of landing and probing. On the other hand, only 22 (2.5%) of the 895 *St. albopicta* examined from the walls were unfed whereas all six culicines were unfed.

## Discussion

*Stegomiya albopicta* females arriving at a human host appear to be positively influenced in their choice of landing site by the recent presence of conspecifics. The *St. albopicta* females started probing within seconds of landing, and most mosquitoes were able to probe before being aspirated by a collector. This may have been sufficient to release chemicals that elicit the observed invitation effect. Despite the fact that roughly a third of the mosquito bites were missed (assuming that the engorged females found on the walls of the shelter had fed in equal proportions on the two collectors), which diluted the detectability of the invitation effect, the effect was strong and significant (randomization test, p < 0.001). This result is in contrast with findings from Ahmadi & McClelland [5], where recently bitten hamsters did not elicit an invitation effect (although the time between being bitten and testing was likely to have exceeded three minutes). A possible alternative explanation for the observed phenomenon in this experiment is that the collectors would be biased in detecting mosquitoes on body parts where they had recently been bitten. However, given that the mosquitoes were collected during the day with good visibility and alert collectors, that the bites caused only mild irritation (c.f. bites of *St. aegypti*), and that mosquitoes were spotted by the collectors on each other as well as on themselves, the strength of the effect, and the fact that the invitation effect has been described previously, such a bias seems unlikely to be the sole explanation for the observed phenomenon. Nevertheless, in future experiments, it would be advisable to collect video evidence of biting activity to control for potential collector bias. This should also allow more exact measures of the distance and time interval at which the effect operates.

Pyrethroids are known to disrupt orientation of mosquitoes towards the host as a result of neural excitement, which appears at an early stage of pyrethroid toxicity [15]. Although the total number of mosquitoes collected on days with pyrethroid was reduced, it did not affect the preference for landing on the most recent previous landing site.

An invitation effect among pool feeding insects can be explained by an increase in efficiency when they feed in a group compared to feeding by individual insects; for example, *Lutziomyia longipalpis*, feeding from the same wound use less saliva to take more blood and produce more eggs than flies that feed as individuals [16]. The advantage of ‘inviting’ other mosquitoes to a host is, however, less readily justifiable. Any chemical produced is likely to be an incidental effect of feeding or probing rather than as a specific signal for other insects, thus it need not be adaptive for the sender and the adaption could be entirely on the part of arriving females since they can be expected to use any and all available stimuli to locate a site on a host suitable for feeding. *Stegomyia albopicta* has a limited flight range (a maximum of 500 m according to [17]). Thus it is likely that many of the insects attracted to a host are relatives. Being able to locate a part of the host that can be fed upon, whilst it may increase the risk of an individual being killed or injured as a result of host defensive behaviour, may enhance the inclusive fitness of a cohort of insects and so be selected for rather than against.

The small number of unfed *St. albopicta* resting on the walls of the shelter implies that most of the mosquitoes that entered landed directly on the host. Although the numbers were very small, the culicines appeared to rest on walls before landing on a host. Hence, inter-current resting [18] prior to feeding may be part of their host location strategy. The structure of the shelter, combined with the lack of inter-current resting in *St. albopicta* may also have affected the body site where the mosquitoes attacked. Clothing may have restricted the number of insects collected landing on the torso of the collectors whilst the head, presumably the nearest body part to a mosquito flying over the wall of the shelter, accounted for 16% of all landings, and by surface area it was the most attacked part of the body. This contrasts with findings reported by Wharton, [19] (quoted in [20]). In that study (in which 282 insects were collected) most mosquitoes attacked the legs and feet (56.2% when standing or 44.4% when lying down) and only 3% of the mosquitoes attacked a person’s head when they were standing or 2.5% when they were lying down.

*Stegomyia albopicta* is an important vector of dengue and chikungunya viruses. It has also been found infected with West Nile, Eastern equine encephalitis, Japanese encephalitis and is a vector of dog heartworm (*Dirofilaria immitis*) [21]. Control of the mosquito is presently the only way of limiting many of these diseases, in particular dengue. The development of traps that use a combination of attractant semio-chemicals is one possibility for control. If the invitation effect is due to a semio-chemical produced by the insect, rather than the result of a reaction by the host to the mosquito bite then, given the low rates of blood fed insects in the sample of landing mosquitoes, it is likely to be produced early in the act of feeding rather than at the time of engorgement. Given the advances in chemical analysis since 1977, the year the invitation ‘effect’ was first described, it should now be possible to determine if a novel chemical is actually released by the mosquitoes, synthesize it and eventually use it in conjunction with other attractants to trap the insects before they get the chance to feed.

## Conclusions

Hungry *Stegomyia albopicta* females appear to respond to recent probing and feeding by conspecifics by landing in the vicinity of sites on the host where this took place. If this effect is due to a semio-chemical produced as a result of feeding it may be possible to identify it as has been done for the airborne aggregation pheromone of the common bed bug, *Cimex lectularius*[22], synthesize it and incorporate it into traps against this important vector of human and animal disease.

## Competing interests

The authors declare that they have no competing interests.

## Authors’ contributions

The collections described in this article were undertaken by JDC and EVET. JDC, L K-H and OJTB analysed the data and wrote the manuscript. All authors read and approved the final manuscript.

## Supplementary Material

Additional file 1 Code used in for statistical analysis in the software package R. Click here for file

Additional file 2 Data. Click here for file
